# (*Z*)-1-(3-Mesityl-3-methyl­cyclo­but­yl)-2-(morpholin-4-yl)ethanone oxime

**DOI:** 10.1107/S1600536811009408

**Published:** 2011-03-23

**Authors:** Fatih Şen, Muharrem Dinçer, Alaaddin Çukurovalı, Ibrahim Yılmaz

**Affiliations:** a Department of Physics, Arts and Sciences Faculty, Ondokuz Mayıs University, 55139 Samsun, Turkey; b Department of Chemistry, Sciences Faculty, Fırat University, 23119 Elazığ, Turkey; c Department of Chemistry, Faculty of Science, Karamanoğlu Mehmetbey University, 70200 Karaman, Turkey

## Abstract

In the title compound, C_20_H_30_N_2_O_2_, the cyclo­butane ring is puckered, with a dihedral angle of 19.60 (13)° between the two planes. In the crystal, the mol­ecules are linked by inter­molecular O—H⋯N and weak C—H⋯O hydrogen bonds, as well as a C—H⋯π hydrogen-bonding association.

## Related literature

For applications of related compounds, see: Dehmlow & Schmidt (1990 ▶); Coghi *et al.* (1976 ▶); Mixich & Thiele (1979 ▶); Migrdichian (1957 ▶); Mathison *et al.* (1989 ▶); Polak (1982 ▶); Balsamo *et al.*, 1990 ▶; Holan *et al.* (1984 ▶); Marsman *et al.* (1999 ▶); Forman (1964 ▶); Bertolasi *et al.* (1982 ▶); Gilli *et al.* (1983 ▶); Hökelek *et al.* (2001 ▶). For related structures, see: Özdemir *et al.* (2004 ▶); Dinçer *et al.* (2004 ▶). For the puckering of the cyclobutane ring, see: Swenson *et al.* (1997 ▶). 
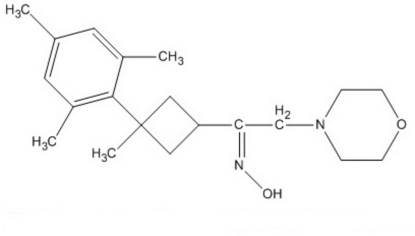


## Experimental

### 

#### Crystal data

C_20_H_30_N_2_O_2_
*M* *_r_* = 330.46
Monoclinic, 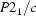

*a* = 13.0273 (4) Å
*b* = 10.2337 (2) Å
*c* = 18.1262 (6) Å
β = 126.574 (2)°
*V* = 1940.69 (10) Å^3^
*Z* = 4
Mo *K*α radiation
μ = 0.07 mm^−1^
*T* = 296 K
0.60 × 0.55 × 0.48 mm

#### Data collection

Stoe IPDS II CCD area-detector diffractometer
Absorption correction: integration (*X-RED32*; Stoe & Cie, 2002 ▶) *T*_min_ = 0.964, *T*_max_ = 0.977
28812 measured reflections
4031 independent reflections
3110 reflections with *I* > 2σ(*I*)
*R*_int_ = 0.058

#### Refinement

*R*[*F* ^2^ > 2σ(*F* ^2^)] = 0.052
*wR*(*F* ^2^) = 0.171
*S* = 1.09
4031 reflections
217 parameters
H-atom parameters constrained
Δρ_max_ = 0.24 e Å^−3^
Δρ_min_ = −0.21 e Å^−3^

### 

Data collection: *X-AREA* (Stoe & Cie, 2002 ▶); cell refinement: *X-AREA*; data reduction: *X-RED32* (Stoe & Cie, 2002 ▶); program(s) used to solve structure: *SHELXS97* (Sheldrick, 2008 ▶); program(s) used to refine structure: *SHELXL97* (Sheldrick, 2008 ▶); molecular graphics: *ORTEP-3 for Windows* (Farrugia, 1997 ▶); software used to prepare material for publication: *WinGX* (Farrugia, 1999 ▶) and *PLATON* (Spek, 2009 ▶).

## Supplementary Material

Crystal structure: contains datablocks global, I. DOI: 10.1107/S1600536811009408/zs2100sup1.cif

Structure factors: contains datablocks I. DOI: 10.1107/S1600536811009408/zs2100Isup2.hkl

Additional supplementary materials:  crystallographic information; 3D view; checkCIF report

## Figures and Tables

**Table 1:** Hydrogen-bond geometry (Å, °) *Cg*1 is the centroid of the benzene ring.

*D*—H⋯*A*	*D*—H	H⋯*A*	*D*⋯*A*	*D*—H⋯*A*
O1—H1⋯N1^i^	0.82	2.11	2.7944 (19)	141
C16—H16*B*⋯O2^ii^	0.97	2.55	3.494 (2)	165
C19—H19*B*⋯O1^iii^	0.97	2.56	3.305 (3)	134
C12—H12*B*⋯*Cg*1^iv^	0.97	2.84	3.777 (3)	161

Symmetry codes: (i) 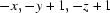; (ii) 
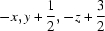
; (iii) 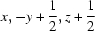; (iv) 
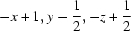
.

**Fig. 1.**
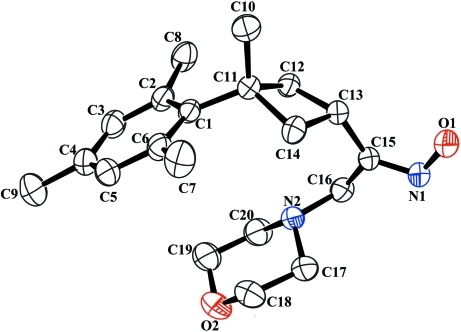
An ORTEP-3 (Farrugia, 1997) drawing of (I), showing the atomic numbering scheme. Displacement ellipsoids are drawn at the 30% probability level. H atoms have been omitted.

**Fig. 2.**
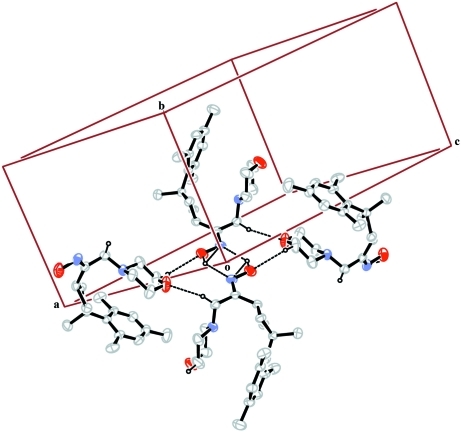
Part of the crystal structure of the title compound, showing the O—H···N and the two C—H···O interactions. For clarity, only H atoms involved in hydrogen bonding have been included. For symmetry codes, see Table 1.

**Fig. 3.**
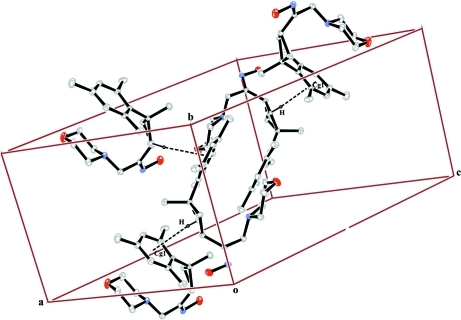
Part of the crystal structure of the title compound, showing the C—H···π interactions. For symmetry codes, see Table 1.

## supplementary crystallographic information

### Comment

It is well known that 3-substituted cyclobutane carboxylic acid derivatives exhibit anti-inflammatory and antidepressant activity (Dehmlow & Schmidt, 1990) and also have liquid crystal properties (Coghi *et al.*, 1976). Oximes show geometric isomerism due to the double bond between the N and C atoms (Mixich & Thiele, 1979; Migrdichian, 1957). As there are significant differences in the physical, chemical and biological properties of these geometric isomers, the determination of the configuration of the isomers is important (Mathison *et al.*, 1989). Oximes and oxime ethers also have a broad pharmacological activity spectrum, encompassing antifungal, antibacterial, antidepressant and insecticidal activities, as well as activity as nerve-gas antidotes, depending on the pharmacophoric group of the molecule (Polak, 1982; Balsamo *et al.*, 1990; Holan *et al.*, 1984; Forman, 1964). The oxime group (C═N—OH) possesses stronger hydrogen-bonding capabilities than the alcohol, phenol or carboxylic acid groups (Marsman *et al.*, 1999). Hydrogen bonding plays a key role in molecular recognition in chemical engineering (Bertolasi *et al.*, 1982; Gilli *et al.*, 1983; Hökelek *et al.*, 2001).

As part of our ongoing study of the relationship between the structures of cyclobutane and oxime derivatives, a crystal structure determination of the title compound C_20_H_30_N_2_O_2_ (I), has been undertaken and the results are presented here. Previously we have reported the crystal structures of similar compounds,*viz.* 2-[2-hydroxyimino-2-(3-methyl-3-phenylcyclobutyl)ethyl]isoindole-1,3-dione, (II) (Özdemir *et al.*, 2004) and 3-[1-hydroxyimino-2-(succinimido)ethyl]-1-methyl-1-phenylcyclobutane, (III) (Dinçer *et al.*, 2004). The main aim of the present investigation was to study the differences among the structures of (I), (II) and (III), and also to determine the strength of the hydrogen-bonding capabilities of the oxime group.

The structure of (I) (Fig. 1) contains a mesityl group (C1–C9), an oxime group (C15,N1,O1), a cyclobutane ring (C11–C14), and a morpholine ring (C17–C20/O2/N2). The mesityl ring comprises an aromatic hydrocarbon with three methyl substituents attached to the benzene ring. The morpholine and cyclobutane rings adopt chair and butterfly conformations respectively. The plane of the morpholine ring forms a dihedral angle of 7.56 (12)° with the plane of the mesityl group and an angle of 47.62 (7)° with the plane of the mesityl ring bonded to atom C11 of the cyclobutane ring. The plane of the cyclobutane ring forms a dihedral angle of 47.86 (8)° with the plane of the morpholine ring.

The C11—C12, C12—C13, C13—C14 and C14—C11 bond lengths are 1.554 (2), 1.533 (2), 1.544 (2) and 1.564 (2) Å respectively and the C11—C12—C13, C11—C14—C13, C12—C11—C14 and C12—C13—C14 bond angles are 91.05 (12), 90.32 (11), 86.85 (11) and 88.33 (11)° respectively within the cyclobutane ring. Although the value for the puckering of the cyclobutane ring found in the literature is 23.5° (Swenson *et al.*, 1997), there is a negligible puckering in the cyclobutane ring in (I): the C11—C12—C13 plane forms a dihedral angle of 20.13 (16)° with the C11—C14—C13 plane while the C14—C13—C12 plane forms a dihedral angle of 19.60 (13)° with the C14—C11—C12 plane of the cyclobutane ring.

In the structure the molecules are linked by an intermolecular oxime O—H···N hydrogen bonds and two weak C—H···O interactions, as well as a C—H···π hydrogen-bonding association (Table 1). These hydrogen bonds link the molecules into infinite chains (Figs. 2 and 3).

### Experimental

A mixture of 10 mmol of 1-mesityl-1-methyl-3-(2-chloro-1-oxoethyl)cyclobutane, 10 mmol of morpholine and 10 mmol of NaHCO_3_ in 30 ml of absolute ethanol was refluxed while monitoring the reaction course using IR techniques. After completion of the reaction, a mixture of 10 mmol of hydroxylammine hydrochloride and 10 mmol of NaOH in 20 ml of absolute ethanol was added portion-wise and refluxed for ten minutes. After cooling to room temperature, the mixture was poured into stirred water. The solid substance thus formed was separated by suction, washed with copious water and recrystallized from ethanol giving white crystals (yield: 68%), m.p. 428 K (EtOH). IR (KBr, ν, cm^-1^): 3287 (–OH), 3089–3024 (aromatics), 2954–2818 (aliphatics), 1612 (C═N), 1483 (C—-N), 1118 (C—O), 939 (N—O); ^1^H NMR (CDCl_3_, TMS, δ, p.p.m.): 1.58 (s, 3H, *p*-CH_3_), 2.32 (s, 6H, *o*-CH_3_s), 2.36 (s, 3H, *p*-CH_3_), 2.40 (m, 4H, –CH_2_– in morpholine ring), 2.59 (d, *J* = 9.6 Hz, 4H, CH_2_– in cyclobutane ring), 3.03 (s, 2H, CH_2_—N), 3.58 (m, 4H, in morpholine ring), 3.65 (quint, *J*_1_ = 7.4 Hz, *J*_2_ = 2.4 Hz, 2H, >CH–, in cyclobutane), 6.77 (s, 2H, aromatics), 8.90 (s, ^1^H, –OH); ^13^C NMR (CDCl_3_, TMS, δ, p.p.m.): 159.31, 144.23, 134.99, 134.60, 130.14, 66.76, 59.08, 53.22, 41.61, 40.98, 28.32, 24.24, 21.36, 20.32.

### Refinement

H atoms were positioned geometrically and treated using a riding model, fixing the bond lengths at 0.96, 0.97, 0.98 and 0.93 Å for CH_3_, CH_2_, CH and CH (aromatic), respectively. The O—H bond length was fixed at 0.93 Å. The displacement parameters of the H atoms were constrained with *U*_iso_(H) = 1.2*U*_eq_(aromatic, methylene or methine C) or 1.5*U*_eq_ (methyl C and oxime O).

### Crystal data

**Table:**

C_20_H_30_N_2_O_2_	*F*(000) = 720
*M**_r_* = 330.46	*D*_x_ = 1.131 Mg m^−^^3^
Monoclinic, *P*2_1_/*c*	Melting point: 428 K
Hall symbol: -P 2ybc	Mo *K*α radiation, λ = 0.71073 Å
*a* = 13.0273 (4) Å	Cell parameters from 29343 reflections
*b* = 10.2337 (2) Å	θ = 1.4–28.0°
*c* = 18.1262 (6) Å	µ = 0.07 mm^−^^1^
β = 126.574 (2)°	*T* = 296 K
*V* = 1940.69 (10) Å^3^	Prism, colourless
*Z* = 4	0.60 × 0.55 × 0.48 mm

### Data collection

**Table:**

Stoe IPDS II CCD area-detector diffractometer	4031 independent reflections
Radiation source: fine-focus sealed tube	3110 reflections with *I* > 2σ(*I*)
graphite	*R*_int_ = 0.058
Detector resolution: 6.67 pixels mm^-1^	θ_max_ = 26.5°, θ_min_ = 2.0°
rotation method scans	*h* = −16→16
Absorption correction: integration (*X-RED32*; Stoe & Cie, 2002)	*k* = −12→12
*T*_min_ = 0.964, *T*_max_ = 0.977	*l* = −22→22
28812 measured reflections	

### Refinement

**Table:**

Refinement on *F*^2^	Primary atom site location: structure-invariant direct methods
Least-squares matrix: full	Secondary atom site location: difference Fourier map
*R*[*F*^2^ > 2σ(*F*^2^)] = 0.052	Hydrogen site location: inferred from neighbouring sites
*wR*(*F*^2^) = 0.171	H-atom parameters constrained
*S* = 1.09	*w* = 1/[σ^2^(*F*_o_^2^) + (0.097*P*)^2^ + 0.1596*P*] where *P* = (*F*_o_^2^ + 2*F*_c_^2^)/3
4031 reflections	(Δ/σ)_max_ < 0.001
217 parameters	Δρ_max_ = 0.24 e Å^−^^3^
0 restraints	Δρ_min_ = −0.21 e Å^−^^3^

### Special details

**Table:**

Geometry. All e.s.d.'s (except the e.s.d. in the dihedral angle between two l.s. planes) are estimated using the full covariance matrix. The cell e.s.d.'s are taken into account individually in the estimation of e.s.d.'s in distances, angles and torsion angles; correlations between e.s.d.'s in cell parameters are only used when they are defined by crystal symmetry. An approximate (isotropic) treatment of cell e.s.d.'s is used for estimating e.s.d.'s involving l.s. planes.
Refinement. Refinement of *F*^2^ against ALL reflections. The weighted *R*-factor *wR* and goodness of fit *S* are based on *F*^2^, conventional *R*-factors *R* are based on *F*, with *F* set to zero for negative *F*^2^. The threshold expression of *F*^2^ > σ(*F*^2^) is used only for calculating *R*-factors(gt) *etc*. and is not relevant to the choice of reflections for refinement. *R*-factors based on *F*^2^ are statistically about twice as large as those based on *F*, and *R*-factors based on ALL data will be even larger.

### Fractional atomic coordinates and isotropic or equivalent isotropic displacement parameters (Å^2^)

**Table:**

	*x*	*y*	*z*	*U*_iso_*/*U*_eq_	
C1	0.36380 (14)	−0.05777 (14)	0.74190 (10)	0.0528 (4)	
C2	0.46281 (15)	−0.05329 (16)	0.83667 (11)	0.0601 (4)	
C3	0.46390 (18)	−0.1424 (2)	0.89474 (12)	0.0715 (5)	
H3	0.5287	−0.1366	0.9575	0.086*	
C4	0.3733 (2)	−0.2391 (2)	0.86371 (15)	0.0758 (5)	
C5	0.28345 (19)	−0.24910 (18)	0.77046 (15)	0.0752 (5)	
H5	0.2242	−0.3167	0.7476	0.090*	
C6	0.27696 (16)	−0.16278 (16)	0.70854 (12)	0.0625 (4)	
C7	0.1786 (2)	−0.1905 (2)	0.60720 (14)	0.0886 (6)	
H7A	0.1295	−0.2660	0.5997	0.133*	
H7B	0.2212	−0.2066	0.5794	0.133*	
H7C	0.1229	−0.1166	0.5782	0.133*	
C8	0.57461 (17)	0.0403 (2)	0.87941 (14)	0.0814 (6)	
H8A	0.5632	0.0968	0.8328	0.122*	
H8B	0.6524	−0.0084	0.9071	0.122*	
H8C	0.5790	0.0918	0.9255	0.122*	
C9	0.3761 (3)	−0.3317 (3)	0.9297 (2)	0.1137 (9)	
H9A	0.4450	−0.3083	0.9916	0.171*	
H9B	0.3885	−0.4194	0.9175	0.171*	
H9C	0.2966	−0.3265	0.9219	0.171*	
C10	0.4242 (2)	0.0076 (2)	0.63806 (16)	0.0834 (6)	
H10A	0.4154	0.0753	0.5979	0.125*	
H10B	0.3894	−0.0724	0.6042	0.125*	
H10C	0.5131	−0.0045	0.6874	0.125*	
C11	0.35215 (14)	0.04679 (15)	0.67740 (11)	0.0556 (4)	
C12	0.38414 (15)	0.18873 (15)	0.71533 (12)	0.0603 (4)	
H12A	0.3857	0.2003	0.7691	0.072*	
H12B	0.4617	0.2227	0.7263	0.072*	
C13	0.26170 (15)	0.24003 (16)	0.62660 (12)	0.0612 (4)	
H13	0.2804	0.2744	0.5853	0.073*	
C14	0.21439 (16)	0.09740 (16)	0.60114 (11)	0.0622 (4)	
H14A	0.1840	0.0734	0.5394	0.075*	
H14B	0.1520	0.0746	0.6119	0.075*	
C15	0.18063 (15)	0.33490 (15)	0.63429 (12)	0.0597 (4)	
C16	0.18719 (17)	0.34202 (17)	0.71954 (13)	0.0659 (4)	
H16A	0.2731	0.3658	0.7713	0.079*	
H16B	0.1291	0.4090	0.7123	0.079*	
C17	0.01643 (17)	0.1917 (2)	0.67605 (14)	0.0768 (5)	
H17A	−0.0136	0.1987	0.6127	0.092*	
H17B	−0.0288	0.2556	0.6863	0.092*	
C18	−0.0090 (2)	0.0568 (3)	0.69414 (19)	0.0989 (7)	
H18A	−0.1000	0.0394	0.6532	0.119*	
H18B	0.0338	−0.0066	0.6813	0.119*	
C19	0.1671 (3)	0.0674 (3)	0.84584 (18)	0.1042 (8)	
H19A	0.2119	0.0042	0.8346	0.125*	
H19B	0.1968	0.0574	0.9089	0.125*	
C20	0.1967 (2)	0.2022 (2)	0.83206 (14)	0.0853 (6)	
H20A	0.1544	0.2658	0.8451	0.102*	
H20B	0.2881	0.2175	0.8738	0.102*	
N1	0.09976 (13)	0.41245 (15)	0.57100 (11)	0.0688 (4)	
N2	0.15302 (12)	0.21666 (14)	0.73755 (9)	0.0607 (4)	
O1	0.09479 (13)	0.40408 (15)	0.49164 (10)	0.0860 (5)	
H1	0.0415	0.4558	0.4531	0.129*	
O2	0.03433 (18)	0.0426 (2)	0.78590 (14)	0.1121 (6)	

### Atomic displacement parameters (Å^2^)

**Table:**

	*U*^11^	*U*^22^	*U*^33^	*U*^12^	*U*^13^	*U*^23^
C1	0.0502 (7)	0.0468 (8)	0.0587 (8)	0.0063 (6)	0.0309 (7)	0.0032 (6)
C2	0.0531 (8)	0.0541 (9)	0.0625 (9)	0.0078 (6)	0.0288 (7)	0.0039 (7)
C3	0.0706 (10)	0.0733 (11)	0.0618 (10)	0.0145 (9)	0.0347 (8)	0.0132 (8)
C4	0.0818 (12)	0.0698 (11)	0.0875 (13)	0.0176 (9)	0.0567 (11)	0.0240 (10)
C5	0.0735 (11)	0.0534 (9)	0.1002 (14)	−0.0039 (8)	0.0526 (11)	0.0056 (9)
C6	0.0595 (9)	0.0489 (8)	0.0701 (10)	−0.0001 (7)	0.0337 (8)	−0.0010 (7)
C7	0.0829 (13)	0.0665 (11)	0.0782 (13)	−0.0119 (9)	0.0273 (10)	−0.0135 (9)
C8	0.0540 (9)	0.0729 (12)	0.0757 (12)	0.0005 (8)	0.0161 (9)	0.0020 (9)
C9	0.132 (2)	0.1057 (19)	0.130 (2)	0.0176 (15)	0.0929 (19)	0.0460 (16)
C10	0.0956 (14)	0.0795 (12)	0.1036 (15)	0.0194 (11)	0.0748 (13)	0.0129 (11)
C11	0.0547 (8)	0.0525 (8)	0.0620 (9)	0.0068 (6)	0.0360 (7)	0.0056 (7)
C12	0.0513 (8)	0.0492 (8)	0.0768 (10)	0.0033 (6)	0.0362 (8)	0.0077 (7)
C13	0.0623 (9)	0.0582 (9)	0.0717 (10)	0.0118 (7)	0.0447 (8)	0.0181 (7)
C14	0.0623 (9)	0.0614 (9)	0.0546 (9)	0.0093 (7)	0.0303 (7)	0.0056 (7)
C15	0.0561 (8)	0.0487 (8)	0.0770 (10)	0.0061 (6)	0.0411 (8)	0.0153 (7)
C16	0.0649 (9)	0.0578 (9)	0.0783 (11)	0.0075 (7)	0.0443 (9)	0.0049 (8)
C17	0.0589 (10)	0.0881 (13)	0.0795 (12)	0.0028 (9)	0.0392 (9)	0.0151 (10)
C18	0.0821 (13)	0.1011 (17)	0.1184 (19)	−0.0112 (12)	0.0623 (14)	0.0161 (14)
C19	0.1032 (17)	0.135 (2)	0.0954 (16)	0.0212 (15)	0.0707 (15)	0.0459 (15)
C20	0.0821 (12)	0.1126 (17)	0.0656 (11)	0.0095 (11)	0.0464 (10)	0.0115 (11)
N1	0.0620 (8)	0.0629 (8)	0.0853 (10)	0.0120 (6)	0.0460 (8)	0.0260 (7)
N2	0.0563 (7)	0.0677 (8)	0.0599 (8)	0.0052 (6)	0.0357 (6)	0.0100 (6)
O1	0.0755 (8)	0.1020 (11)	0.0870 (9)	0.0268 (7)	0.0519 (7)	0.0429 (8)
O2	0.1121 (13)	0.1290 (15)	0.1348 (15)	0.0072 (10)	0.0949 (12)	0.0415 (11)

### Geometric parameters (Å, °)

**Table:**

C1—C2	1.406 (2)	C12—H12A	0.9700
C1—C6	1.409 (2)	C12—H12B	0.9700
C1—C11	1.524 (2)	C13—C15	1.501 (2)
C2—C3	1.386 (3)	C13—C14	1.544 (2)
C2—C8	1.515 (3)	C13—H13	0.9800
C3—C4	1.378 (3)	C14—H14A	0.9700
C3—H3	0.9300	C14—H14B	0.9700
C4—C5	1.371 (3)	C15—N1	1.271 (2)
C4—C9	1.509 (3)	C15—C16	1.498 (3)
C5—C6	1.391 (3)	C16—N2	1.457 (2)
C5—H5	0.9300	C16—H16A	0.9700
C6—C7	1.514 (3)	C16—H16B	0.9700
C7—H7A	0.9600	C17—N2	1.453 (2)
C7—H7B	0.9600	C17—C18	1.500 (3)
C7—H7C	0.9600	C17—H17A	0.9700
C8—H8A	0.9600	C17—H17B	0.9700
C8—H8B	0.9600	C18—O2	1.410 (3)
C8—H8C	0.9600	C18—H18A	0.9700
C9—H9A	0.9600	C18—H18B	0.9700
C9—H9B	0.9600	C19—O2	1.414 (3)
C9—H9C	0.9600	C19—C20	1.493 (4)
C10—C11	1.533 (2)	C19—H19A	0.9700
C10—H10A	0.9600	C19—H19B	0.9700
C10—H10B	0.9600	C20—N2	1.456 (2)
C10—H10C	0.9600	C20—H20A	0.9700
C11—C12	1.554 (2)	C20—H20B	0.9700
C11—C14	1.563 (2)	N1—O1	1.404 (2)
C12—C13	1.533 (2)	O1—H1	0.8200
			
C2—C1—C6	117.83 (14)	H12A—C12—H12B	110.8
C2—C1—C11	120.93 (14)	C15—C13—C12	118.29 (15)
C6—C1—C11	121.24 (14)	C15—C13—C14	117.48 (14)
C3—C2—C1	119.55 (16)	C12—C13—C14	88.30 (12)
C3—C2—C8	117.12 (16)	C15—C13—H13	110.3
C1—C2—C8	123.24 (16)	C12—C13—H13	110.3
C4—C3—C2	122.95 (17)	C14—C13—H13	110.3
C4—C3—H3	118.5	C13—C14—C11	90.31 (12)
C2—C3—H3	118.5	C13—C14—H14A	113.6
C5—C4—C3	116.83 (17)	C11—C14—H14A	113.6
C5—C4—C9	122.0 (2)	C13—C14—H14B	113.6
C3—C4—C9	121.2 (2)	C11—C14—H14B	113.6
C4—C5—C6	122.97 (18)	H14A—C14—H14B	110.9
C4—C5—H5	118.5	N1—C15—C16	114.07 (15)
C6—C5—H5	118.5	N1—C15—C13	124.80 (17)
C5—C6—C1	119.40 (16)	C16—C15—C13	121.10 (13)
C5—C6—C7	117.43 (17)	N2—C16—C15	110.55 (14)
C1—C6—C7	123.12 (16)	N2—C16—H16A	109.5
C6—C7—H7A	109.5	C15—C16—H16A	109.5
C6—C7—H7B	109.5	N2—C16—H16B	109.5
H7A—C7—H7B	109.5	C15—C16—H16B	109.5
C6—C7—H7C	109.5	H16A—C16—H16B	108.1
H7A—C7—H7C	109.5	N2—C17—C18	108.85 (16)
H7B—C7—H7C	109.5	N2—C17—H17A	109.9
C2—C8—H8A	109.5	C18—C17—H17A	109.9
C2—C8—H8B	109.5	N2—C17—H17B	109.9
H8A—C8—H8B	109.5	C18—C17—H17B	109.9
C2—C8—H8C	109.5	H17A—C17—H17B	108.3
H8A—C8—H8C	109.5	O2—C18—C17	111.6 (2)
H8B—C8—H8C	109.5	O2—C18—H18A	109.3
C4—C9—H9A	109.5	C17—C18—H18A	109.3
C4—C9—H9B	109.5	O2—C18—H18B	109.3
H9A—C9—H9B	109.5	C17—C18—H18B	109.3
C4—C9—H9C	109.5	H18A—C18—H18B	108.0
H9A—C9—H9C	109.5	O2—C19—C20	110.93 (19)
H9B—C9—H9C	109.5	O2—C19—H19A	109.5
C11—C10—H10A	109.5	C20—C19—H19A	109.5
C11—C10—H10B	109.5	O2—C19—H19B	109.5
H10A—C10—H10B	109.5	C20—C19—H19B	109.5
C11—C10—H10C	109.5	H19A—C19—H19B	108.0
H10A—C10—H10C	109.5	N2—C20—C19	109.40 (19)
H10B—C10—H10C	109.5	N2—C20—H20A	109.8
C1—C11—C10	111.39 (13)	C19—C20—H20A	109.8
C1—C11—C12	116.03 (14)	N2—C20—H20B	109.8
C10—C11—C12	111.90 (15)	C19—C20—H20B	109.8
C1—C11—C14	116.62 (13)	H20A—C20—H20B	108.2
C10—C11—C14	111.97 (15)	C15—N1—O1	113.21 (15)
C12—C11—C14	86.87 (11)	C17—N2—C20	109.05 (15)
C13—C12—C11	91.08 (12)	C17—N2—C16	112.40 (13)
C13—C12—H12A	113.5	C20—N2—C16	113.32 (16)
C11—C12—H12A	113.5	N1—O1—H1	109.5
C13—C12—H12B	113.5	C18—O2—C19	109.51 (16)
C11—C12—H12B	113.5		
			
C6—C1—C2—C3	−6.9 (2)	C11—C12—C13—C15	134.65 (15)
C11—C1—C2—C3	173.84 (15)	C11—C12—C13—C14	14.10 (13)
C6—C1—C2—C8	169.57 (16)	C15—C13—C14—C11	−135.28 (15)
C11—C1—C2—C8	−9.6 (2)	C12—C13—C14—C11	−14.01 (13)
C1—C2—C3—C4	1.8 (3)	C1—C11—C14—C13	131.49 (14)
C8—C2—C3—C4	−174.94 (18)	C10—C11—C14—C13	−98.55 (16)
C2—C3—C4—C5	3.5 (3)	C12—C11—C14—C13	13.83 (13)
C2—C3—C4—C9	−178.3 (2)	C12—C13—C15—N1	159.02 (16)
C3—C4—C5—C6	−3.6 (3)	C14—C13—C15—N1	−97.0 (2)
C9—C4—C5—C6	178.3 (2)	C12—C13—C15—C16	−22.9 (2)
C4—C5—C6—C1	−1.6 (3)	C14—C13—C15—C16	81.1 (2)
C4—C5—C6—C7	175.97 (19)	N1—C15—C16—N2	118.59 (16)
C2—C1—C6—C5	6.9 (2)	C13—C15—C16—N2	−59.7 (2)
C11—C1—C6—C5	−173.91 (15)	N2—C17—C18—O2	59.1 (2)
C2—C1—C6—C7	−170.59 (17)	O2—C19—C20—N2	−59.6 (3)
C11—C1—C6—C7	8.6 (3)	C16—C15—N1—O1	−179.84 (14)
C2—C1—C11—C10	91.37 (19)	C13—C15—N1—O1	−1.6 (2)
C6—C1—C11—C10	−87.81 (19)	C18—C17—N2—C20	−58.0 (2)
C2—C1—C11—C12	−38.2 (2)	C18—C17—N2—C16	175.45 (18)
C6—C1—C11—C12	142.60 (15)	C19—C20—N2—C17	58.7 (2)
C2—C1—C11—C14	−138.40 (15)	C19—C20—N2—C16	−175.29 (17)
C6—C1—C11—C14	42.4 (2)	C15—C16—N2—C17	−74.95 (18)
C1—C11—C12—C13	−132.15 (13)	C15—C16—N2—C20	160.85 (15)
C10—C11—C12—C13	98.51 (15)	C17—C18—O2—C19	−59.2 (3)
C14—C11—C12—C13	−13.94 (13)	C20—C19—O2—C18	59.2 (3)

### Hydrogen-bond geometry (Å, °)

**Table:**

*D*—H···*A*	*D*—H	H···*A*	*D*···*A*	*D*—H···*A*
O1—H1···N1^i^	0.82	2.11	2.7944 (19)	141
C16—H16B···O2^ii^	0.97	2.55	3.494 (2)	165
C19—H19B···O1^iii^	0.97	2.56	3.305 (3)	134
C12—H12B···Cg1^iv^	0.97	2.84	3.777 (3)	161

Symmetry codes: (i) −*x*, −*y*+1, −*z*+1; (ii) −*x*, *y*+1/2, −*z*+3/2; (iii) *x*, −*y*+1/2, *z*+1/2; (iv) −*x*+1, *y*−1/2, −*z*+1/2.
